# *Cannabis* microbiome sequencing reveals several mycotoxic fungi native to dispensary grade *Cannabis* flowers

**DOI:** 10.12688/f1000research.7507.2

**Published:** 2016-05-10

**Authors:** Kevin McKernan, Jessica Spangler, Lei Zhang, Vasisht Tadigotla, Yvonne Helbert, Theodore Foss, Douglas Smith

**Affiliations:** 1 Medicinal Genomics Corporation, Woburn, MA, USA

**Keywords:** Cannabis, Microbiome, Mycotoxins, Cannabidiol, Paxilline, Citrinin, qPCR, Culture, Next generation sequencing

## Abstract

The Center for Disease Control estimates 128,000 people in the U.S. are hospitalized annually due to food borne illnesses. This has created a demand for food safety testing targeting the detection of pathogenic mold and bacteria on agricultural products. This risk extends to medical *Cannabis* and is of particular concern with inhaled, vaporized and even concentrated *Cannabis *products *.* As a result, third party microbial testing has become a regulatory requirement in the medical and recreational *Cannabis* markets, yet knowledge of the *Cannabis* microbiome is limited. Here we describe the first next generation sequencing survey of the fungal communities found in dispensary based *Cannabis* flowers by ITS2 sequencing, and demonstrate the sensitive detection of several toxigenic *Penicillium *and *Aspergillus *species, including *P. citrinum and P. paxilli, *that were not detected by one or more culture-based methods currently in use for safety testing.

## Introduction

Our knowledge of the natural microbiome of field-grown *Cannabis* in terms of rhizosphere bacteria, and endophytic fungi is limited to just a few focused studies ^1– 3^. Very little is known about the potential for bacterial and fungal contamination on medicinal *Cannabis*. Nevertheless, many states in the U.S. are now crafting regulations for detection of microbial contamination on *Cannabis* in the absence of any comprehensive survey of actual samples. A few of these regulations are inducing growers to “heat kill” or pasteurize *Cannabis* flowers to lower microbial content. While this seems a harmless suggestion, we must remain aware of how these drying techniques may create false negatives in culture-based safety tests used to monitor colony-forming units (CFU). Even though pasteurization may be effective at sterilizing some of the microbial content, it does not eliminate various pathogenic toxins or spores. *Aspergillus* spores and mycotoxins are known to resist pasteurization ^4, 5^. Similar thermal resistance has been reported for *E. coli* produced Shiga toxin ^6^. While pasteurization may reduce CFU’s used in petri-dish or plating based safety tests, it does not reduce the microbial toxins, spores or DNA encoding these toxins.

Monitoring for mycotoxic fungi in cannabis preparations has been recommended as part of routine safety testing by the Cannabis Safety Institute. A major driver for this recommendation has been numerous reported cases of serious or fatal pulmonary Aspergillosis associated with marijuana smoking in immunocompromised patients ^7– 9^. The major cannabinoids have been shown to be potent inhibitors of several cytochrome P450 enzymes at therapeutic concentrations, including 1A1, 1A2, 1B1 2B6, 2C19, 2D6, 3A4 and 3A5 ^10^. Some of these enzymes have been implicated in the metabolism of the fungal toxins aflatoxin and ochratoxin ^11– 13^. This raises questions about potential interactions and appropriate safety tolerances for mycotoxins in patients being treated with cannabinoid therapeutics. In addition, some *Fusarium* species that produce toxins have proven to be difficult to selectively culture with tailored media ^14– 16^. This is a common problem associated with culture-based systems as carbon sources are not exclusive to certain microbes and only 1% of microbial species are believed to be culturable ^17^.

While the risks of mycotoxic fungal contamination have been well studied in the food markets, the presence of the fungal populations present on *Cannabis* flowers has never been surveyed with next generation sequencing techniques ^18– 23^. With the publication of the *Cannabis* genome ^24, 25^ and many other pathogenic microbial genomes, quantitative PCR assays have been developed that can accurately quantify fungal DNA present in *Cannabis* samples ^26^. Here, we analyze the yeast and mold species present in 10 real world, dispensary-derived *Cannabis* samples by quantitative PCR and sequencing, and demonstrate the presence of several mycotoxin producing fungal strains that are not detected by widely used culture-based assays.

## Methods

### Culture-based methods

The culture-based methods selected for testing here represent those currently in use by established medicinal *Cannabis* safety testing laboratories. 3.55ml of tryptic soy broth (TSB) was used to wet 250mg of homogenized flower in a whirlpack bag. TSB was aspirated from the reverse side of the 100μm mesh filter and placed into a Biolumix ^TM^ growth vial and spread onto a 3M Petri Film ^TM^ and a SimPlate ^TM^ (3M Petrifilm ^TM^ 3M Microbiology, St. Paul, MN, USA; SimPlates ^TM^ Biocontrol Systems, Bellevue, WA, USA; BioLumix ^TM^ Neogen, Lansing MI, USA) according to the respective manufacturers’ recommendations. Biolumix ^TM^ vials were grown and monitored for 48 hours while Petri-films ^TM^ and SimPlates ^TM^ were grown for 5 days. Petri-films ^TM^ and SimPlates ^TM^ were colony counted manually by three independent observers. Samples were tested on total coliform, total entero, total aerobic, and total yeast and mold. Only total yeast and mold discrepancies were graduated to sequencing.

### DNA purification

Plant DNA was extracted with SenSATIVAx according to manufacturers’ instructions (Medicinal Genomics part #420001). DNA was eluted with 50μl ddH20.

### Primers used for PCR and sequencing

PCR was performed using 5μl of DNA (3ng/μl) 12.5μl 2X LongAmp (NEB) with 1.25μl of each 10μM MGC-ITS3 and MGC-ITS3 primer (MGC-ITS3; TACACGACGTTGTAAAACGACGCATCGATGAAGAACGCAGC) and (MGC-ITS3R; AGGATAACAATTTCACACAGGATTTGAGCTCTTGCCGCTTCA) with 10μl ddH20 for a 25μl total reaction. An initial 95°C 5 minute denaturization was performed followed by 40 cycles of 95°C for 15s and 65°C for 90s. Samples were purified with 75μl SenSATIVAx, washed twice with 100μl 70% EtOH and bench dried for 5 minutes at room temperature. Samples were eluted in 25μl ddH20.

### Total Yeast and Mold assay and ITS amplification

A commercially available total yeast and mold qPCR assay (TYM-PathogINDICAtor, Medicinal Genomics, Woburn MA) was used to screen for fungal DNA in a background of host *Cannabis* DNA. The TYM qPCR assay targets the ribosomal DNA Internal Transcribed Spacer region 2 (ITS2) using modified primers described previously ^27, 28^. Fungal DNA amplified using these primers may also be subjected to next generation sequencing to identify the contributing yeast and mold species. ITS sequencing has been widely used to identify and enumerate fungal species present in a given sample ^29^.

### Tailed PCR cloning and sequencing

DNA libraries were constructed with 250ng DNA using New England Biolabs (Ipswich, MA) NEBNext Quick ligation module (NEB # E6056S). End repair used 3μl of enzyme mix, 6.5μl of reagent mix, 55.5μl of DNA + ddH20. Reaction was incubated at 30°C for 20 minutes. After end repair, ligation was performed directly with 15μl of blunt end TA mix, 2.5μl of Illumina adaptor (10μM) and 1μl of ligation enhancer (assumed to be 20% PEG 6000). After 15 minute ligation at 25°C, 3μl of USER enzyme was added to digest the hairpin adaptors and prepare for PCR. The USER enzyme was tip-mixed and incubated at 37°C for 20 minutes. After USER digestion, 86.5μl of SenSATIVAx was added and mixed. The samples were placed on a magnet for 15 minutes until the beads cleared and the supernatant could be removed. Beads were washed twice with 150μl of 70% EtOH. Beads were left for 10 minute to air dry and then eluted in 25μl of 10mM Tris-HCl.

### Library PCR

25μl 2X Q5 polymerase was added to 23μl of DNA with 1μl of i7 index primer (25μM) and 1μl universal primer (25μm). After an initial 95°C for 10s, the library was amplified for 15 cycles of 95°C 10s, 65°C 90s. Samples were purified by mixing 75μl of SenSATIVAx into the PCR reaction. The samples were placed on a magnet for 15 minutes until the beads cleared and the supernatant could be removed. Beads were washed twice with 150μl of 70% EtOH. Beads were left for 10 minute to air dry and then eluted in 25μl of 10mM Tris-HCl. Samples were prepared for sequencing on the MiSeq version 2 chemistry according to the manufacturers’ instructions. 2×250bp reads were selected to obtain maximal ITS sequence information.

### PaxP verification PCR

Primers described by Shirazi-zand *et al*. were utilized to amplify a segment of the 725bp *PaxP* gene from *Penicillium paxilli*. 25μl LongAmp (NEB) 4μl 10μM primer, 1μl DNA (14ng/μl), 20μl ddH20 to make a 50μl PCR reaction. Cycling conditions were slightly modified to accommodate a different polymerase. 95°C for 30s followed by 28 cycles of 95°C 15s, 55°C for 30s, 65°C 2.5 minutes. Samples were purified with 50μl of SenSATIVAx as described above. 1μl of purified PCR product was sized on Agilent HS 2000 chip. Nextera libraries and sequencing were performed according to instructions from Illumina using 2×75bp sequencing on a version 2 MiSeq.

### *Penicillium Citrinum* verification PCR

Citrinum forward GATTTTCCAAAATGCCGTCT and Citrinum reverse GCTCAAGCATTAATCTAGCTA primers were used with identical PCR conditions as above with the exception using 35 cycles of PCR. Samples were purified with 50μl of SenSATIVAx as described above. 1μl of purified PCR product was sized on Agilent HS 2000 chip. Nextera libraries and sequencing were performed according to instructions from Illumina using 2×75bp sequencing on a version 2 MiSeq. Reads were mapped to Genbank accession number LKUP01000000. Mappings were confirmed using BLAST to NCBI to ensure the strongest hits were to *P. citrinum*.

### Analysis

Reads were demultiplexed and trimmed with Casava 1.8.2 and trim_galore v0.4.1 ( http://www.bioinformatics.babraham.ac.uk/projects/trim_galore/). FLASH v1.2.11 ^30^ was used to merge the reads using max_overlap 150. The reads were aligned to microbial references using MG-RAST v3.2 ^31^. Alignments and classifications were confirmed with a second software tool from One Codex ( https://onecodex.com/) and critical pathways identified for further evaluation with PCR of toxin producing genes. Reads are deposited in NCBI under SRA accession: SRP065410. Nextera 2×75bp sequencing of the *PaxP* gene was mapped to accession number HM171111.1 with CLCbio Workstation V4 at 98% identity over 80% of the read. One Codex analysis was put into Public mode under the following public URLs:

Australian Bastard:

https://app.onecodex.com/analysis/public/201e7f1642e04a3c

https://app.onecodex.com/analysis/public/58f1e03c10434bfa

KD4:

https://app.onecodex.com/analysis/public/2e86e262817246c4

https://app.onecodex.com/analysis/public/1abd5b60446140a0

KD6:

https://app.onecodex.com/analysis/public/a92d3dff5485499d

https://app.onecodex.com/analysis/public/8d72e2514e564ecd

KD8:

https://app.onecodex.com/analysis/public/8d72e2514e564ecd

https://app.onecodex.com/analysis/public/d6e2e0bcfba3469f

Liberty Haze:

https://app.onecodex.com/analysis/public/7bcd650fa5544f2c

https://app.onecodex.com/analysis/public/7f0feb6cb0a94d56

Girls Scout Cookie:

https://app.onecodex.com/analysis/public/a71b1ce8331c461d

https://app.onecodex.com/analysis/public/8d6f10c7ee684f93

Jakes Grape:

https://app.onecodex.com/analysis/public/bc8af5ed19e5407a

https://app.onecodex.com/analysis/public/99d7a4a2f7af486b

RECON:

https://app.onecodex.com/analysis/public/8a22a16cc2e24731

https://app.onecodex.com/analysis/public/0af6ae26a01f48d5

GreenCrack:

https://app.onecodex.com/analysis/public/6114843d2eb3425e

https://app.onecodex.com/analysis/public/3eee642786c54a88

LA Confidential:

https://app.onecodex.com/analysis/public/01e8aefb0d4f4f62

https://app.onecodex.com/analysis/public/b74c2988fcd84e38

NYC Diesel:

https://app.onecodex.com/analysis/public/441cfad759f64dcc

https://app.onecodex.com/analysis/public/d97b39cae96c4a44

## Results

**Figure 1.**
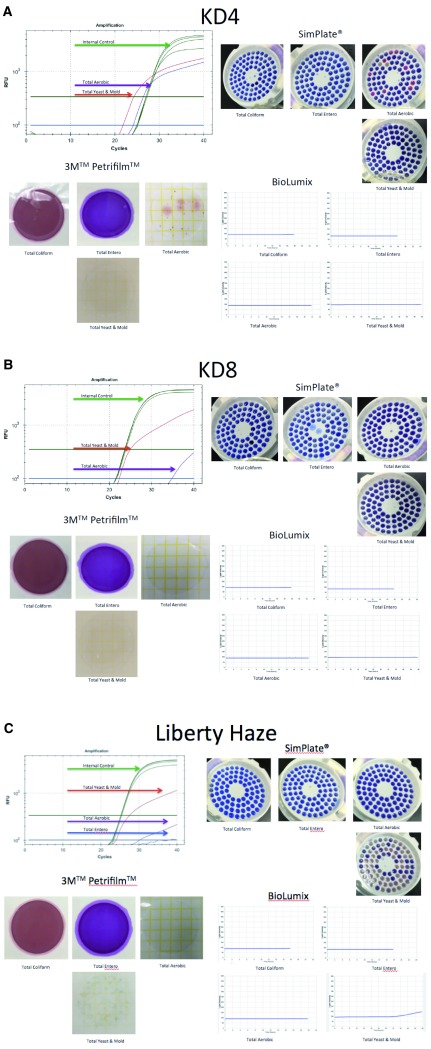
Comparison of 4 different microbial detection technologies. **Figure 1A**. qPCR signal from TYM (red line) test run concurrently (multiplexed) with a plant internal control marker (green line). This marker targets a conserved region in the *Cannabis* genome and should show up in every assay (upper left). SimPlates count the number of discolored wells (purple to pink) as a proxy for CFU/gram. Only total aerobic show growth (upper right). Petrifilm only demonstrate colonies on total aerobic platings (lower left). Biolumix demonstrate no signal across all 4 tests (lower right). **Figure 1B**. Sample KD8 fails to culture any total yeast and mold yet demonstrates significant TYM qPCR signal. Sample was graduated to ITS based next generation sequencing. **Figure 1C**. Sample Liberty Haze was tested with 3 culture based methods and compared to qPCR. Sample was graduated to ITS based next generation sequencing.

We purified DNA from *Cannabis* samples obtained from two different geographic regions (Amsterdam and Massachusetts) several years apart (2011 and 2015). The majority of samples purified and screened with ITS qPCR were negative for amplification signal implying reagents clean of fungal contamination *.* Six of the 17 dispensary-derived *Cannabis* samples tested positive for yeast and mold in the TYM qPCR assay. These results were compared with the results derived from three commercially available culture-based detection systems for each of the 17 samples (3M Petrifilm ^TM^ 3M Microbiology, St. Paul, MN, USA; SimPlates ^TM^ Biocontrol Systems, Bellevue, WA, USA; BioLumix ^TM^ Neogen, Lansing MI, USA; Figure 1). Of the 6 qPCR positive samples, two tested negative in all 3 culture-based assays and four tested negative in 1 or 2 of the culture-based assays ( Table 1). None of the qPCR negative samples tested positive in any of the culture-based assays. Each of the 6 discordant samples was subjected to ITS sequencing to precisely identify the collection of microbes present. Four additional samples from a different geographic origin (Amsterdam) were also subjected to ITS sequencing, for a total of 10 *Cannabis* samples.

**Table 1.** Samples were cultured with 3 different techniques and compared to quantitative PCR (qPCR). Biolumix had the lowest sensitivity failing to pick up 4/17 samples detected with other culture-based platforms. qPCR identified 2 samples that were not picked up by any other method. Positive qPCR samples were sequenced to identify the contributing signal. Highlighted samples fail the 10,000 CFU/g cutoffs which equates to a Cq of 26 on the qPCR assay according to the manufacturers’ instructions. (f) is fail or over 10,000 CFU/g. (p) is pass or under 10,000 CFU/g. The raw CFU numbers can be deduced by dividing the CFU number by the 1,000 fold dilution factor used in this study.

Samples	Total Yeast and Mold (10,000 CFU/g = fail)			
	Simplate ^®^ (CFU/g)	3M ^®^ (CFU/g)	BioLumix ^®^ (CFU/g)	Cq
**KD4**	0	0	pass	21.71 (f)
**KD8**	0	0	pass	22.5 (f)
PC3	0	0	pass	>40 (p)
White Widow	0	0	pass	>40 (p)
KD1	0	0	pass	29.33 (p)
KD2	0	0	pass	>40 (p)
KD3	0	0	pass	30.16 (p)
KD5	1000 (p)	6000 (p)	pass	27.76 (p)
KD6	3000 (p)	19000 (f)	pass	24.72 (f)
KD7	0	0	pass	>40 (p)
Liberty Haze	172000 (f)	89000 (f)	pass	24.02 (f)
Blueberry Kush	0	0	pass	37.99 (p)
Blueberry Kush -spiked	>738,000 (f)	TNTC (f)	fail	15.71 (f)
Girl Scout Cookies	>738,000 (f)	TNTC (f)	pass	19.66 (f)
Jake's Grape	>738,000 (f)	TNTC (f)	pass	24.56 (f)
Serious Happiness	0	0	pass	>40 (p)
White Rhino	0	3000 (p)	pass	>40 (p)

TNTC = Too Numerous To Count

**Figure 2.**
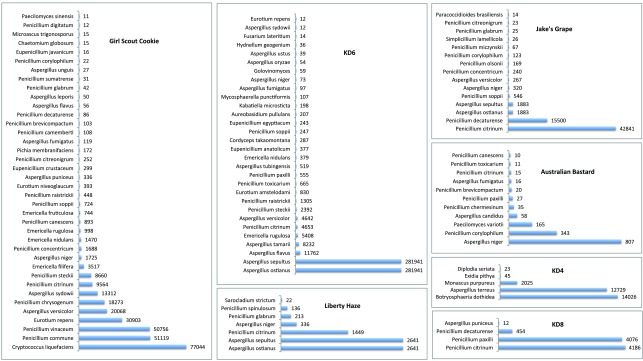
Detection of fungal species by ITS2 sequencing and MG-RAST analysis. Histograms are provided for each of the *Cannabis* samples that tested negative by at least one culture based method and positive using a qPCR-based total yeast and mold test. The number of reads corresponding to each detected fungal species is indicated to the right of each bar. Species detected with less than 10 reads are not included. The overall read counts per sample are more a reflection of sample normalization for sequencing than of the absolute fungal DNA levels.

Each discordant sample presented with an array of microbial species, as shown in Figure 2. No sample presented with a single dominant species, and each sample displayed multiple species of interest. Of particular concern were the identified DNA sequences from toxin producing species: *Aspergillus versicolor* ^32– 36^, *Aspergillus terreus* ^37^, *Penicillium citrinum* ^38– 40^, *Penicillium paxilli* ^41, 42^.

**Figure 3.**
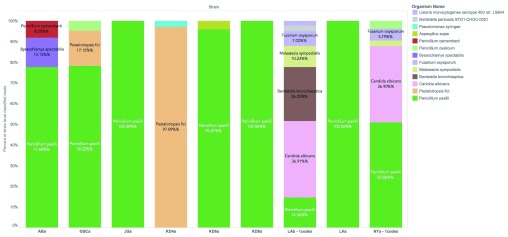
One Codex classification of ITS reads. *P. paxilli* is the most frequently found contaminant in *Cannabis* flowers. *P. citrinum* is not in the One Codex database at this time. One Codex utilizes a fast k-mer based approach for whole genome shotgun classification and can be influenced by read trimming and database content. The reads provided to MG-RAST were trimmed and FLASH’d (paired end reads merged when overlapping) prior to classification. K-mer based approaches can significantly differ from longer word size methods and this underscores the importance of confirmatory PCR in microbiome analysis.

**Figure 4.**
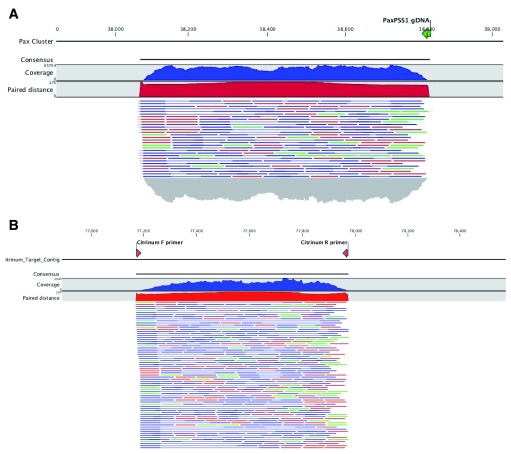
PCR of genes encoding Paxilline and Citrinin demonstrates amplification of the expected size. Citrinum primers we designed from Genbank accession number LKUP01000753. Paxilline primers were used as described in Saikia *et al*. PCR products were made into shotgun libraries with Nextera and sequenced on an Illumina MiSeq with 2×75bp reads to over 10,000X coverage. Reads were mapped with CLCbio 4 to NCBI accession number HM171111.1 ( **A**) and LKUP01000000 respectively ( **B**). Paired reads are displayed as blue lines, green and red lines are unpaired reads. Read coverage over the amplicons are depicted in a blue histogram over the cluster while paired end read distance is measured in a red histogram over the region. Off target read mapping is limited. *P. paxilli* mappings are displayed on top ( **A**) and *P. citrinum* mappings are displayed on bottom ( **B**). Alignment of PCR primers to *P. paxilli* reference shows a 5 prime mismatch that is a result of the primers being designed to target spliced RNA according to Saikia *et al.*

We further analyzed the ITS sequence alignments using the whole genome shotgun based microbiome classification software known as One Codex ^43^. Nine of the ten samples sequenced showed the presence of *P. paxilli* ( Figure 3). To verify the accuracy of this ITS phylotyping, a gene involved in the paxilline toxin biosynthesis pathway of *P. paxilli* was amplified with PaxPss1 and PaxPss2 primers described by Saikia *et al.* ^44^. The resulting 725bp amplicon (expected size) was sequenced to confirm the presence of the *P. paxilli* biosynthesis gene in the *Cannabis* sample KD8 ( Figure 4). This was successfully repeated with primers designed to target genes in the citrinin pathway of *P. citrinum*. There were some discrepancies between the results derived from the two software platforms (One Codex and MG-RAST). The MG-RAST analysis, using merged, paired reads correlated better with the PCR results. While One Codex predicted and confirmed KD8 as having the highest *P. paxilli* content, the One Codex platform is optimized for whole genome shotgun data and may not be able to differentiate the 18S sequence differences (391/412 aligned bases) between these two species with a K-mer based approach.

With the confirmed presence of *P. paxilli*, we are curious to find out whether the toxin, paxilline, is present in the samples. Development of monoclonal antibodies to paxilline has recently been described ^45^, but commercial ELISA assays with sensitivity under 50ppb do not appear to be available at this time. A >50ppb multiplexed ELISA assay is available from Randox Food Diagnostics (Crumlin, UK). Detection with LC-MS/MS has also been described ^46, 47^, however, and experiments are underway to determine whether paxilline can be identified in the background of cannabinoids and terpenes present in *Cannabis* samples.

## Discussion

This study demonstrates detection of numerous fungal species by molecular screening of ITS2 in several dispensary-derived *Cannabis* samples. These included the toxigenic *Penicillium* species: *P. paxilli, P. citrinum*, *P. commune, P. chrysogenum, P. corylophilum, Aspergillus* species: *A. terreus, A. niger, A. flavus*, *A. versicolor* and *Eurotium repens.* In addition, a pathogenic species *Cryptococcus liquefaciens* was detected. The fungal microbiomes of the different samples differed significantly in the number and diversity of species present. Two samples contained a large diversity of species, similar to previous studies that used field-grown samples and culture-based outgrowth methods ^2, 3, 48^. Other samples contained only a few species at significant levels. This is perhaps not surprising given the prevalence of indoor culture methods using artificial growth media for medicinal *Cannabis*. However, we do not have any knowledge of the specific growth conditions that were used for the samples analyzed.

Three different culture-based assays failed to detect all of the positive samples and one, BioLumix ^TM^, detected only one out of 7 positive samples. A review of the literature suggests that *Penicillium* microbes can be cultured on CYA media, but some may require colder temperatures (21-24C) and 7 day growth times ^49^. Of the *Penicillium,* only *P. citrinum* has been previously reported to culture with 3M Petri-Film ^50^. It is possible the different water activity of the culture assay compared to the natural flower environment is contributing to the false negative test results.

Quantitative PCR is agnostic to water activity and can be performed in hours instead of days. The specificity and sensitivity provides important information on samples that present risks invisible to culture based systems. The drawback to qPCR is the method’s indifference to living or non-living DNA. While techniques exist to perform live-dead qPCR, the live status of the microbes is unrelated to toxin potentially produced while the microbes were alive. ELISA assays exist to screen for some toxins ^51^. Current state-recommended ELISA’s do not detect citrinin or paxilline, the toxins produced by *P. citrinum* and *P. paxilli*, respectively. The predominance of these *Penicillium* species in a majority of the samples tested is interesting. Several *Penicillium* species are known to be endophytes on various plant species, including *P. citrinum* ^18^, and this raises the question of whether they may be common *Cannabis* endophytes. Indeed, *P. citrinum* and a species identified as *P. copticola* (a member of the citrinun section ^51^) have previously been identified as *Cannabis* endophytes, along with several *Aspergillus* species ^2, 3^.

Paxilline is a tremorgenic and ataxic potassium channel blocker and has been shown to attenuate the anti-seizure properties of cannabidiol in certain mouse models ^52– 54^. Paxilline is reported to have tremorgenic effects at nanomolar concentrations and is responsible for Ryegrass-staggers disease ^55^. Cannabidiol is often used at micromolar concentrations for seizure reduction and contamination with paxilline, if confirmed, would be a cause for concern. Citrinin is a mycotoxin that disrupts Ca2+ efflux in the mitochondrial permeability transition pore (mPTP) ^56– 63^. Ryan *et al.* demonstrated that cannabidiol affects this pathway suggesting a similar potential cause for concern regarding CBD-citrinin interaction ^64^. Considering the hydrophobicity of these mycotoxins and the growing interest in the use of extracted oils from CBD-rich *Cannabis* strains for treatment of drug resistant epilepsy ^65– 70^, more precise molecular screening of fungal toxins in these products might be warranted.

ITS amplification and sequencing offers a hypothesis-free testing approach that can be employed to identify a broad range of fungal species present in a given sample. Appropriate primer design can survey a broad spectrum of fungal genomes while affording rapid iteration of design. Quantitative PCR has also demonstrated single molecule sensitivity and linear dynamic range over 5 orders of magnitude offering a very sensitive approach for detection of microbial risks. Our survey of *Cannabis* flowers in this study was limited, however. Further studies are required to survey a broader range of samples, and to determine whether paxilline, citrinin, aflatoxin or ochratoxin can be detected at concentrations that represent a clinical risk in *Cannabis* samples or extracts derived from plants that test positive for the fungi known to produce those toxins.

## Conclusions

Several toxigenic fungi were detected in dispensary-derived *Cannabis* samples using molecular amplification and sequencing techniques. These microbes were not detected using traditional culture-based platforms. These results suggest that culture based techniques borrowed from the food industry should be re-evaluated for *Cannabis* testing to ensure that they are capable of detecting the prevalent species detected by molecular methods with adequate sensitivity. We recommend that additional sequencing studies be performed to characterize the fungal and bacterial microbiomes of a more diverse selection of *Cannabis* samples. Such sampling should include dispensary-derived samples from both indoor and outdoor crops, as well as samples from police seizures from well-provenanced foreign sources, such as Mexico. Finally, further studies should be performed to measure toxin levels in strains that test positive for toxigenic species.
