# Supplementary material for: Cannabis microbiome sequencing reveals several mycotoxic fungi native to dispensary grade Cannabis flowers
Source: F1000Res. 2016 May 10;4:1422. Originally published 2015 Dec 10. [Version 2] doi: 10.12688/f1000research.7507.2 (PMC4897766; doi:10.12688/f1000research.7507.2)
Supplement: Peer review history

# Peer review: Referee response for version 2

**DOI:** 10.5256/f1000research.9041.r13979

**Published:** 2016-06-06

**Authors:** Donald Cooper

**Affiliations:** 1 Chief Science Officer, Mobile Assay Inc., Boulder, CO, USA

The authors have adequately addressed most of my concerns with the exception of the issue of re-evaluating the standard methods used for establishing food safety thresholds as applied to cannabis.  

 The problem is with the use of the word "re-evaluate".  The authors are suggesting that the negative results they obtained using cell culture-based platforms in samples that were positive using their more sensitive molecular amplification indicate a possible limitation of standard methods as applied to cannabis. This may or may not be true. The concern I have is whether their technique is overly sensitive based on current limits used in the food industry. It may be that use of molecular DNA amplification methods indicate the presence of several toxigenic species of fungi and other pathogens in food but at such low levels that standard culture-based methods would not detect them. In this case there would be no need to re-evaluate the use of standard methods that establish safety thresholds. In short, without some type of calibration between the author's technique and traditional methods there is no way of knowing whether their sensitivity is too high or traditional methods used in the food industry are somehow not capable of detecting pathogens in cannabis preparations and therefore need to be "re-evaluated".

I have read this submission. I believe that I have an appropriate level of expertise to confirm that it is of an acceptable scientific standard.

# Peer review: Referee response for version 1

**DOI:** 10.5256/f1000research.8086.r11800

**Published:** 2016-02-04

**Authors:** Donald Cooper

**Affiliations:** 1 Chief Science Officer, Mobile Assay Inc., Boulder, CO, USA

The manuscript is well written and appropriate as an article in F1000 Research. The abstract states that their findings, "demonstrate the limitations in the culture-based regulations", but this conclusion does not follow from their data. In fact, their results show that their DNA based method is overly sensitive at detecting potential pathogens. Whether culture-based regulations are appropriate or not would have to be validated and tested directly, not simply inferred from the presence of microbial DNA. The authors should remove this cautionary sentence in the abstract and throughout the manuscript until it has been validated. This would make the manuscript more balanced and justified.

A better understanding of the microbiome and mycobiome in cannabis is an important endeavor in part, because recent work on the human microbiome has revealed that microbial constituents of the microbiome and fungi interact cooperatively and non cooperatively to influence human health. Recently studies focused on the human gut mycobiome have been performed using deep sequencing of the ITS1 region for identification of fungi in fecal samples from healthy individuals and the researchers identified 184 fungal species in total. Human oral and lung tissues testing indicate the presence of *Aspergillus, Cryptococcus, Fusarium*, and *Alternaria *in healthy individuals. An emerging theme from this new field of study indicates complex microbial communities distributed across the body that fundamentally contribute to the development, physiology and metabolic homeostasis of the macro-organism. The same is likely true in plants, like cannabis. Because of this beneficial dynamic interplay between microbes and hosts a complete absence of amplified DNA or RNA microbial markers would be unexpected. The question is, “What levels are safe?” and the answer to this question has not been established.

From a consumer safety perspective the rationale for microbial testing in food and pharmaceuticals is to prevent infection of highly toxigenic microbes that grow readily and are consumed in rather large quantities. For example, *Aspergillus flavus* infects grain in as much as 30% of Sub-Saharan African maize and the Aflatoxin regulatory limit for maize is 10 ppb because in this region maize is consumed in kilogram quantities on a daily basis. The risk of health hazard to individuals arising from microbial toxins or mycotoxins is largely proportional to the consumption amounts and frequency. Compared to other regulated foods or tobacco it is expected that the level of cannabis consumption would be minimal and would therefore present minimal risk, nevertheless, there is burgeoning interest in putting in place regulatory requirements for medical and recreational Cannabis in some markets.  

In this present study the authors use state-of-the art technology to identify DNA-based markers associated with a variety of microbes and, as expected, some are pathogenic. The authors state that “Mycotoxin monitoring in Cannabis preparations is important since aflatoxin produced by *Aspergillus* species is a carcinogen.”, but there is no actual data showing that the strain of *Aspergillus* they identify is toxigenic. *Aspergillus* is a common fungus that is found in the human mycobiome in oral, lung, gastrointestinal tract. Detection of *Aspergillus *markers in minute quantities alone is not necessarily a health concern unless it is also coincident with live cells that can grow readily and secrete toxin that are above some threshold or there is an imbalance in homeostatic growth limiting factors. The authors call into question culture-based testing, which is the standard in Food safety and USP regulatory guidelines based on comparison to their highly sensitive DNA based detection. Their results using standard regulatory methods, for the most part, would not indicate a food safety problem. The authors should add this emphasis. The authors correctly identify a limitation of their study in that the qPCR based testing has an “indifference to living or non-living DNA” and because of this their PCR based approach may be unnecessarily sensitive. To date there are little to no guidelines for thresholds on many mycotoxins or bacterial toxins that have been established in the cannabis industry, so their findings help inform regulators as to which type of toxins might be relevant for further analysis. 

The authors state, ”Health compromised patients exposed to aflatoxin and clearance-inhibiting cannabinoids raise new questions in regards to the current safety tolerances to aflatoxin.” but the authors present no data showing the presence of aflatoxin in any of their samples. So their caution is highly speculative and they should indicate that unless they provide data supporting their caution.  

As mentioned above, dynamic homeostatic processes limit the growth of microbes and fungi in living organisms and the authors point out that “several studies have demonstrated plant phytochemicals and terpenoids like eugenol can inhibit the growth of fungi. It is possible the different water activity of the culture assay compared to the natural terpene rich flower environment is contributing to the false negative test results.”  but these phytochemicals may also prevent the growth of fungi and bacteria in the plant despite the presence of microbial DNA. 

Furthermore they state that “While techniques exist to perform live-dead qPCR, the live status of the microbes is unrelated to toxin potentially produced while the microbes were alive. ELISA assays exist to screen for some toxins. Current state-recommended ELISA’s do not detect citrinin or paxilline, the toxins produced by P. citrinum and P. paxilli, respectively. The predominance of these Penicillium species in a majority of the samples tested is interesting.” Ideally the authors would test for these toxins in their most positive samples.

The authors state that, “Cannabidiol is often used at micromolar concentrations for seizure reduction implying sub-percentage contamination of paxilline could still be a concern” but this is highly speculative and the authors should de-emphasize the “concern” and state instead that if their results were verified by tests indicating high levels of paxilline then it may be cause for concern. The same is true for their concern about Citrinin and aflatoxin and the authors should state this. 

The authors state, “While ELISA assays are easy point of use tests that can be used to detect fungal toxins, they can suffer from lack of sensitivity and cross reactivity. ITS amplification and sequencing offers hypothesis-free testing that can complement the lack of specificity in ELISA assays.” ELISA and rapid diagnostic lateral flow tests are standard in the food safety industry for measuring toxins. I see no need to call into question protein based ELISA methods without even testing them in the first place. All diagnostic tests have sensitivity and selectivity limitations which is why they need to be tested and verified using other analytical methods. 

The authors state that “Appropriate primer design can survey a broad spectrum of microbial genomes while affording rapid iteration of design. Quantitative PCR has also demonstrated single molecule sensitivity and linear dynamic range over 5 orders of magnitude offering a very robust approach for detection of microbial risks. This may be important for the detection of nanomolar potency mycotoxins”. The ability to detect single copies of genes makes their system highly sensitive, but does not indicate level of mycotoxin. The authors should point out the limitations of their approach and discuss the possibility that it would likely generate a high degree of false positive results compared to culture-based standard methods. 

The authors state, “These results demonstrate that culture based techniques superimposed from the food industry should be re-evaluated based on the known microbiome of actual Cannabis flowers in circulation at dispensaries.”  This statement appears to be too strong in light of their data. Without validation for the presence of toxins above a safety threshold there is no need to re-evaluate the standard methods in the food industry.

I have read this submission. I believe that I have an appropriate level of expertise to confirm that it is of an acceptable scientific standard, however I have significant reservations, as outlined above.

# Response

**Published:** 2016-03-17

**Authors:** Kevin Mckernan

**Affiliations:** Medicinal Genomics Corporation, USA

*The manuscript is well written and appropriate as an article in F1000 Research. The abstract states that their findings, "demonstrate the limitations in the culture-based regulations", but this conclusion does not follow from their data. In fact, their results show that their DNA based method is overly sensitive at detecting potential pathogens. Whether culture-based regulations are appropriate or not would have to be validated and tested directly, not simply inferred from the presence of microbial DNA. The authors should remove this cautionary sentence in the abstract and throughout the manuscript until it has been validated. This would make the manuscript more balanced and justified.*

Author response: The last sentence of the abstract was revised to read: Here we describe the first next generation sequencing survey of the fungal communities found in dispensary based *Cannabis* flowers by ITS2 sequencing, and demonstrate the sensitive detection of several toxigenic *Penicillium *and *Aspergillus *species, including *P. citrinum and P. paxilli, *that were not detected by one or more culture-based methods currently in use for safety testing.

*A better understanding of the microbiome and mycobiome in cannabis is an important endeavor in part, because recent work on the human microbiome has revealed that microbial constituents of the microbiome and fungi interact cooperatively and non cooperatively to influence human health. Recently studies focused on the human gut mycobiome have been performed using deep sequencing of the ITS1 region for identification of fungi in fecal samples from healthy individuals and the researchers identified 184 fungal species in total. Human oral and lung tissues testing indicate the presence of Aspergillus, Cryptococcus, Fusarium, and Alternaria in healthy individuals. An emerging theme from this new field of study indicates complex microbial communities distributed across the body that fundamentally contribute to the development, physiology and metabolic homeostasis of the macro-organism. The same is likely true in plants, like cannabis. Because of this beneficial dynamic interplay between microbes and hosts a complete absence of amplified DNA or RNA microbial markers would be unexpected. The question is, “What levels are safe?” and the answer to this question has not been established.*

*From a consumer safety perspective the rationale for microbial testing in food and pharmaceuticals is to prevent infection of highly toxigenic microbes that grow readily and are consumed in rather large quantities. For example, Aspergillus flavusinfects grain in as much as 30% of Sub-Saharan African maize and the Aflatoxin regulatory limit for maize is 10 ppb because in this region maize is consumed in kilogram quantities on a daily basis. The risk of health hazard to individuals arising from microbial toxins or mycotoxins is largely proportional to the consumption amounts and frequency. Compared to other regulated foods or tobacco it is expected that the level of cannabis consumption would be minimal and would therefore present minimal risk, nevertheless, there is burgeoning interest in putting in place regulatory requirements for medical and recreational Cannabis in some markets.*

*In this present study the authors use state-of-the art technology to identify DNA-based markers associated with a variety of microbes and, as expected, some are pathogenic. The authors state that “Mycotoxin monitoring in Cannabis preparations is important since aflatoxin produced by Aspergillus species is a carcinogen.”, but there is no actual data showing that the strain of Aspergillus they identify is toxigenic. Aspergillus is a common fungus that is found in the human mycobiome in oral, lung, gastrointestinal tract. Detection of Aspergillus markers in minute quantities alone is not necessarily a health concern unless it is also coincident with live cells that can grow readily and secrete toxin that are above some threshold or there is an imbalance in homeostatic growth limiting factors. The authors call into question culture-based testing, which is the standard in Food safety and USP regulatory guidelines based on comparison to their highly sensitive DNA based detection. Their results using standard regulatory methods, for the most part, would not indicate a food safety problem. The authors should add this emphasis. The authors correctly identify a limitation of their study in that the qPCR based testing has an “indifference to living or non-living DNA” and because of this their PCR based approach may be unnecessarily sensitive. To date there are little to no guidelines for thresholds on many mycotoxins or bacterial toxins that have been established in the cannabis industry, so their findings help inform regulators as to which type of toxins might be relevant for further analysis.*

*The authors state, ”Health compromised patients exposed to aflatoxin and clearance-inhibiting cannabinoids raise new questions in regards to the current safety tolerances to aflatoxin.” but the authors present no data showing the presence of aflatoxin in any of their samples. So their caution is highly speculative and they should indicate that unless they provide data supporting their caution.*

**

Author response: We thank the reviewer for the background and context provided to his concern. The questions about what levels of toxins are safe or acceptable fall well beyond the scope of the present study.  We concede, however, that some statements made in the introduction may have been too speculative.  We have revised paragraph 2 of the introduction to focus on the numerous literature reports of pulmonary aspergillosis associated with cannabis use instead of potential mycotoxin toxicity. We have also provided more background on the overlap of cannabinoid and mycotoxin metabolism via cytochrome P450 system as follows.  Monitoring for mycotoxic fungi in cannabis preparations has been recommended as part of routine safety testing by the Cannabis Safety Institute. A major driver for this recommendation has been numerous reported cases of serious or fatal pulmonary Aspergillosis associated with marijuana smoking in immunocompromised patients ^4-6^. The major cannabinoids have been shown to be potent inhibitors of several cytochrome P450 enzymes at therapeutic concentrations, including 1A1, 1A2, 1B1 2B6, 2C19, 2D6, 3A4 and 3A5 ^7^. Some of these enzymes have been implicated in the metabolism of the fungal toxins aflatoxin and ochratoxin ^8-10^. This raises questions about potential interactions and appropriate safety tolerances for mycotoxins in patients being treated with cannabinoid therapeutics. In addition, some *Fusarium* species that produce toxins have proven to be difficult to selectively culture with tailored media ^6^^– ^^8^ . This is a common problem associated with culture-based systems as carbon sources are not exclusive to certain microbes and only 1% of microbial species are believed to be culturable ^9^ .

The first sentence of the next paragraph was edited slightly to follow more logically:  While the risks of mycotoxic fungal contamination have been well studied in the food markets, the presence of the fungal populations present on *Cannabis* flowers has never been surveyed with next generation sequencing techniques ^10^^– ^^15^ .

*As mentioned above, dynamic homeostatic processes limit the growth of microbes and fungi in living organisms and the authors point out that “several studies have demonstrated plant phytochemicals and terpenoids like eugenol can inhibit the growth of fungi. It is possible the different water activity of the culture assay compared to the natural terpene rich flower environment is contributing to the false negative test results.”  but these phytochemicals may also prevent the growth of fungi and bacteria in the plant despite the presence of microbial DNA.*

Author response: Upon reconsideration, we concluded that there is no way to predict how growth of the specific fungi detected might be affected by the terpenoids in Cannabis flowers. Those compounds may inhibit the growth of some fungi, while permitting the growth of others. In any case, the effect of terpenes would not relevant to the ability of the fungi to grow on culture media lacking those compounds. That sentence was deleted and the last sentence of paragraph 1 in the discussion was edited as follows:  It is possible the different water activity of the culture assay compared to the natural flower environment is contributing to the false negative test results.

*Furthermore they state that “While techniques exist to perform live-dead qPCR, the live status of the microbes is unrelated to toxin potentially produced while the microbes were alive. ELISA assays exist to screen for some toxins. Current state-recommended ELISA’s do not detect citrinin or paxilline, the toxins produced by P. citrinum and P. paxilli, respectively. The predominance of these Penicillium species in a majority of the samples tested is interesting.” Ideally the authors would test for these toxins in their most positive samples.*

Author response: We stated transparently that this needed to be done as a future direction. We don’t see it as an essential part of the current publication, which had the goal of simply cataloging the fungal content of dispensary-derived samples.

*The authors state that, “Cannabidiol is often used at micromolar concentrations for seizure reduction implying sub-percentage contamination of paxilline could still be a concern” but this is highly speculative and the authors should de-emphasize the “concern” and state instead that if their results were verified by tests indicating high levels of paxilline then it may be cause for concern. The same is true for their concern about Citrinin and aflatoxin and the authors should state this.*

*We agree. Paragraph 3 of the discussion was edited to address this criticism as follows:  Paxilline is a tremorgenic and ataxic potassium channel blocker and has been shown to attenuate the anti-seizure properties of cannabidiol in certain mouse models ^44^^– ^^46^ . Paxilline is reported to have tremorgenic effects at nanomolar concentrations and is responsible for Ryegrass-staggers disease ^47^ . Cannabidiol is often used at micromolar concentrations for seizure reduction and contamination with paxilline, if confirmed, would be a cause for concern. Citrinin is a mycotoxin that disrupts Ca2+ efflux in the mitochondrial permeability transition pore (mPTP) ^48^^– ^^55^ . Ryan et al. demonstrated that cannabidiol affects this pathway suggesting a similar potential cause for concern regarding CBD-citrinin interaction ^56^ . Considering the hydrophobicity of these mycotoxins and the growing interest in the use of extracted oils from CBD-rich Cannabis strains for treatment of drug resistant epilepsy ^57^^– ^^62^, more precise molecular screening of fungal toxins in these products might be warranted.*

*The authors state, “While ELISA assays are easy point of use tests that can be used to detect fungal toxins, they can suffer from lack of sensitivity and cross reactivity. ITS amplification and sequencing offers hypothesis-free testing that can complement the lack of specificity in ELISA assays.” ELISA and rapid diagnostic lateral flow tests are standard in the food safety industry for measuring toxins. I see no need to call into question protein based ELISA methods without even testing them in the first place. All diagnostic tests have sensitivity and selectivity limitations which is why they need to be tested and verified using other analytical methods.*

*The authors state that “Appropriate primer design can survey a broad spectrum of microbial genomes while affording rapid iteration of design. Quantitative PCR has also demonstrated single molecule sensitivity and linear dynamic range over 5 orders of magnitude offering a very robust approach for detection of microbial risks. This may be important for the detection of nanomolar potency mycotoxins”. The ability to detect single copies of genes makes their system highly sensitive, but does not indicate level of mycotoxin. The authors should point out the limitations of their approach and discuss the possibility that it would likely generate a high degree of false positive results compared to culture-based standard methods.*

Author response: The comments about ELISA assays was deleted and the paragraph was edited to focus on detection of fungal species, not toxins, as follows.  ITS amplification and sequencing offers a hypothesis-free testing approach that can be employed to identify a broad range of fungal species present in a given sample. Appropriate primer design can survey a broad spectrum of fungal genomes while affording rapid iteration of design. Quantitative PCR has also demonstrated single molecule sensitivity and linear dynamic range over 5 orders of magnitude offering a very sensitive approach for detection of microbial risks. Our survey of *Cannabis* flowers in this study was limited, however. Further studies are required to survey a broader range of samples, and to determine whether paxilline, citrinin, aflatoxin or ochratoxin can be detected at concentrations that represent a clinical risk in *Cannabis* samples or extracts derived from plants that test positive for the fungi known to produce those toxins.

*The authors state, “These results demonstrate that culture based techniques superimposed from the food industry should be re-evaluated based on the known microbiome of actual Cannabis flowers in circulation at dispensaries.”  This statement appears to be too strong in light of their data. Without validation for the presence of toxins above a safety threshold there is no need to re-evaluate the standard methods in the food industry.*

Author response: We respectfully disagree with this comment of the reviewer. The sentence was taken out of context.  We were not trying to suggest that the standard methods in use in the food industry should be re-evaluated for all applications, only the use of those methods for medicinal *Cannabis* testing. The Conclusions paragraph was modified to clarify as follows. Several toxigenic fungi were detected in dispensary-derived *Cannabis* samples using molecular amplification and sequencing techniques. These microbes were not detected using traditional culture-based platforms. These results suggest that culture based techniques borrowed from the food industry should be re-evaluated for *Cannabis* testing to ensure that they are capable of detecting the prevalent species detected by molecular methods with adequate sensitivity. We recommend that additional sequencing studies be performed to characterize the fungal and bacterial microbiomes of a more diverse selection of *Cannabis *samples. Such sampling should include dispensary-derived samples from both indoor and outdoor crops, as well as samples from police seizures from well-provenanced foreign sources, such as Mexico. Finally, further studies should be performed to measure toxin levels in strains that test positive for toxigenic species.

# Peer review: Referee response for version 1

**DOI:** 10.5256/f1000research.8086.r11516

**Published:** 2015-12-17

**Authors:** John McPartland

**Affiliations:** 1 Division of Molecular Biology,, GW Pharmaceutical, Salisbury, UK

This succinct, well-written study has two major aims: 1. Utilize NextGen and qPCR to identify microorganisms inhabiting dispensary-sourced *Cannabis *flowers. 2. Compare results from these sequencing techniques to results from traditional culture-based methods. Results from the qPCR survey led to a third aim: confirm the presence of two heretofore unreported mycotoxin-producing fungi on *Cannabis: Penicillium citrinum* and *Penicillium paxilli*.

Five critiques:

1. “Microbiome” appears in the manuscript’s title, so you should cite some literature regarding plant microbiomes in general, as well as *Cannabis*-specific research. Vorholt (2012) and Turner *et al.* (2013) provide general overviews. *Cannabis*-specific microbiome studies (Kusari *et al.* 2013, Gautam *et al.* 2013) generated very different results than yours, and should be discussed. The rhizosphere study by Winston *et al.* (2014) ought to be mentioned, and highlight the complimentary nature of rhizosphere and phyllosphere studies.

      The foliar microbiome ( *aka*, phyllosphere, as opposed to rhizosphere) can be partitioned into two groups: *epiphytes* live upon the leaf epidermis, and *endophytes* occupy intercellular spaces within the leaf. Culture-based detection systems normally surface-sterilize plant samples, so they assume that cultured organisms are endophytes. NextGen and qPCR should identify both epiphytes and endophytes. Classic epiphytes identified in your study include *Kabatiella (Aureobasidium) microsticta* and *Sarocladium (Acremonium) strictum*.

      Phyllosphere organisms may be plant pathogens, and cause disease symptoms; diseased plants should never reach a dispensary. However, phyllosphere organisms may act as symbionts (good for the plant) or commensals (indifferent), and their asymptomatic presence is not easily detected. Nevertheless these cryptic organisms may cause disease in humans. The spores from phyllosphere fungi readily pass through waterpipes (Moody *et al.* 1982), and survive in smoke drawn from cannabis cigarettes (Kurup *et al. *1983), as do aflatoxins (Llewellyn and O'Rear 1977). Worth mentioning.

2. Explain the methodology used to select three culture-based detection systems in this study. Are they the most widely-used systems? Are they the systems recommended by cannabis regulatory agencies? If the method was simply “convenience sampling,” say so.

3. Methods used in the qPCR ITS assay should be described in the Methods section, not the Results section.

4. Figure 2, “DNA sequencing of ITS2 amplicons from culture negative samples that are qPCR positive for total yeast and mold tests,” deserves some comment. Some of the taxa are not yeasts or molds. They include angiosperms *(Zea mays, Pachysandra procumbens*), a protozoan ( *Sterkiella histriomuscorum*), and an “uncultured bacterium.” Comment please.

5. This study revealed a surprisingly depauperate *Cannabis* foliar microbiome, compared to a recent study of *Genlisea* species, using similar methods, that identified 92 genera of organisms (Cao *et al.* 2015). See Delmotte *et al.* (2009) for rich microbiomes in other plant species. Gzebenyuk (1984) isolated 79 species of fungi from hemp stems in Russia. Comment please.

      Where are the bacteria? Much of the concern over microbiology and food safety focuses on human enteric pathogens ( *e.g., Escherichia coli, Salmonella* spp.) and opportunistic bacteria ( *e.g., Pseudomonas aeruginosa, Berkholderia cepacia*). Previous culture-based studies have isolated these organisms from *Cannabis* ( *e.g.,* Taylor *et al.* 1982, Ungerleider *et al. *1982). The only bacterium you identify (in Figure 2, erroneously identified as a yeast or mold) is *Pantoea agglomerans, *formerly known as *Enterobacter agglomerans,* a gram-negative bacterium and an opportunistic human pathogen.

Minor critiques:

1. The detection and confirmation of *Penicillium citrinum* and *Penicillium paxilli* deserves mention in the abstract of the paper! An exhaustive review of the *Cannabis* literature (McPartland *et al.* 2000) found no references to these organisms.
2. Species names should never be capitalized. For example, *P. Citrinum* should read *P. citrinum*.  
3. The full name should be spelled out the first time it appears in the Methods section, *Penicillium citrinum*.
4. The Methods section should explain that the *PaxP* gene comes from *Penicillium paxilli.* The Methods section should identify NEP as New England BioLabs.
5. Recommend some future directions: a comparison of indoor crops and outdoor crops (outdoor crops may show a seasonal community succession), and survey the microbiome of police seizures from well-provenanced foreign sources, such as Mexico.

I have read this submission. I believe that I have an appropriate level of expertise to confirm that it is of an acceptable scientific standard.

## References

[1] CaoHXSchmutzerTScholzUPecinkaASchubertIVuGT: Metatranscriptome analysis reveals host-microbiome interactions in traps of carnivorous Genlisea species. *Front Microbiol*.2015;6: 10.3389/fmicb.2015.00526 526 10.3389/fmicb.2015.00526 26236284PMC4500957

[2] DelmotteNKniefCChaffronSInnerebnerGRoschitzkiBSchlapbachRvon MeringCVorholtJA: Community proteogenomics reveals insights into the physiology of phyllosphere bacteria. *Proc Natl Acad Sci U S A*.2009;106(38) : 10.1073/pnas.0905240106 16428-33 10.1073/pnas.0905240106 19805315PMC2738620

[3] GautamAKantMThakurY: Isolation of endophytic fungi fromCannabis sativa and study their antifungal potential. *Archives Of Phytopathology And Plant Protection*.2013;46(6) : 10.1080/03235408.2012.749696 627-635 10.1080/03235408.2012.749696

[4] GzebenyukNV: The occurrence of fungi on hemp stems. *Mikologiya i Fitopathologiya*.1984;18(4) :322-326

[5] KurupVPResnickAKagenSLCohenSHFinkJN: Allergenic fungi and actinomycetes in smoking materials and their health implications. *Mycopathologia*.1983;82(1) :61-4 634854810.1007/BF00436948

[6] KusariPKusariSSpitellerMKayserO: Endophytic fungi harbored in Cannabis sativa L.: diversity and potential as biocontrol agents against host plant-specific phytopathogens. *Fungal Diversity*.2013;60(1) : 10.1007/s13225-012-0216-3 137-151 10.1007/s13225-012-0216-3

[7] LlewellynGCO'RearCE: Examination of fungal growth and aflatoxin production on marihuana. *Mycopathologia*.1977;62(2) :109-12 41413810.1007/BF01259400

[8] McPartlandJMClarkeRCWatsonDP: Hemp diseases and pests. *CABI Publishing (Wallingford;UK)*.2000;

[9] MoodyMMWhartonRCSchnaperNSchimpffSC: Do water pipes prevent transmission of fungi from contaminated marijuana?. *N Engl J Med*.1982;306(24) :1492-3 7078603

[10] TaylorDNWachsmuthIKShangkuanYHSchmidtEVBarrettTJSchraderJSScherachCSMcGeeHBFeldmanRABrennerDJ: Salmonellosis associated with marijuana: a multistate outbreak traced by plasmid fingerprinting. *N Engl J Med*.1982;306(21) : 10.1056/NEJM198205273062101 1249-53 10.1056/NEJM198205273062101 7070444

[11] TurnerTRJamesEKPoolePS: The plant microbiome. *Genome Biol*.2013;14(6) : 10.1186/gb-2013-14-6-209 209 10.1186/gb-2013-14-6-209 23805896PMC3706808

[12] UngerleiderJTAndrysiakTTashkinDPGaleRP: Contamination of marihuana cigarettes with pathogenic bacteria--possible source of infection in cancer patients. *Cancer Treat Rep*.1982;66(3) :589-91 7060050

[13] VorholtJA: Microbial life in the phyllosphere. *Nat Rev Microbiol*.2012;10(12) : 10.1038/nrmicro2910 828-40 10.1038/nrmicro2910 23154261

[14] WinstonMEHampton-MarcellJZarraonaindiaIOwensSMMoreauCSGilbertJAHartselJAHartselJKennedySJGibbonsSM: Understanding cultivar-specificity and soil determinants of the cannabis microbiome. *PLoS One*.2014;9(6) : 10.1371/journal.pone.0099641 e99641 10.1371/journal.pone.0099641 24932479PMC4059704

# Response

**Published:** 2016-03-17

**Authors:** Kevin Mckernan

**Affiliations:** Medicinal Genomics Corporation, USA

This succinct, well-written study has two major aims: 1. Utilize NextGen and qPCR to identify microorganisms inhabiting dispensary-sourced *Cannabis *flowers. 2. Compare results from these sequencing techniques to results from traditional culture-based methods. Results from the qPCR survey led to a third aim: confirm the presence of two heretofore unreported mycotoxin-producing fungi on *Cannabis: Penicillium citrinum* and *Penicillium paxilli*.

Five critiques:

1. “Microbiome” appears in the manuscript’s title, so you should cite some literature regarding plant microbiomes in general, as well as *Cannabis*-specific research. Vorholt (2012) and Turner *et al.* (2013) provide general overviews. *Cannabis*-specific microbiome studies (Kusari *et al.* 2013, Gautam *et al.* 2013) generated very different results than yours, and should be discussed. The rhizosphere study by Winston *et al.* (2014) ought to be mentioned, and highlight the complimentary nature of rhizosphere and phyllosphere studies.

      The foliar microbiome ( *aka*, phyllosphere, as opposed to rhizosphere) can be partitioned into two groups: *epiphytes*live upon the leaf epidermis, and *endophytes* occupy intercellular spaces within the leaf. Culture-based detection systems normally surface-sterilize plant samples, so they assume that cultured organisms are endophytes. NextGen and qPCR should identify both epiphytes and endophytes. Classic epiphytes identified in your study include *Kabatiella (Aureobasidium) microsticta* and *Sarocladium (Acremonium) strictum*.

      Phyllosphere organisms may be plant pathogens, and cause disease symptoms; diseased plants should never reach a dispensary. However, phyllosphere organisms may act as symbionts (good for the plant) or commensals (indifferent), and their asymptomatic presence is not easily detected. Nevertheless these cryptic organisms may cause disease in humans. The spores from phyllosphere fungi readily pass through waterpipes (Moody *et al.* 1982), and survive in smoke drawn from cannabis cigarettes (Kurup *et al. *1983), as do aflatoxins (Llewellyn and O'Rear 1977). Worth mentioning.

**Author response: Several of the references cited relate to analysis of the bacterial microbiome on diverse plant species, which falls well beyond the scope of this study (focusing on the fungal microbiome of *Cannabis*).  However, we added a brief mention of the very limited literature relating to Cannabis microbiomes to the introduction as follows:**

"Our knowledge of the natural microbiome of field-grown *Cannabis *in terms of rhizosphere bacteria, and endophytic fungi is limited to just a few focused studies ^1-3^. Very little is known about the potential for bacterial and fungal contamination on medicinal *Cannabis*. Nevertheless, many states in the U.S. are now crafting regulations for detection of microbial contamination on *Cannabis* in the absence of any comprehensive survey of actual samples. A few of these regulations are inducing growers to “heat kill” or pasteurize *Cannabis* flowers to lower microbial content. While this seems a harmless suggestion, we must remain aware of how these drying techniques may create false negatives in culture-based safety tests used to monitor colony-forming units (CFU). Even though pasteurization may be effective at sterilizing some of the microbial content, it does not eliminate various pathogenic toxins or spores. *Aspergillus* spores and mycotoxins are known to resist pasteurization ^1^^, ^^2^ . Similar thermal resistance has been reported for *E. coli* produced Shiga toxin ^3^ . While pasteurization may reduce CFU’s used in petri-dish or plating based safety tests, it does not reduce the microbial toxins, spores or DNA encoding these toxins."

**Author response: We also added a sentence to the end of the second paragraph of the discussion in reference to the two existing publications on Cannabis endophytic fungi.**

"Several *Penicillium* species are known to be endophytes on various plant species, including *P. citrinum* ^10^ , and this raises the question of whether they are also *Cannabis* endophytes. Indeed, *P. citrinum* and a species identified as *P. copticola* (a member of the citrinun clade ^12^) have previously been identified as *Cannabis* endophytes, along with several *Aspergillus* species ^2, 3^."

2. Explain the methodology used to select three culture-based detection systems in this study. Are they the most widely-used systems? Are they the systems recommended by cannabis regulatory agencies? If the method was simply “convenience sampling,” say so.

**Author response: A sentence was added to the culture based methods section.  **The culture-based methods selected for testing here represent those currently in use by established medicinal Cannabis safety testing laboratories.

3. Methods used in the qPCR ITS assay should be described in the Methods section, not the Results section.

**Author response: Paragraph 1 of the results section was moved to the methods section.**

4. Figure 2, “DNA sequencing of ITS2 amplicons from culture negative samples that are qPCR positive for total yeast and mold tests,” deserves some comment. Some of the taxa are not yeasts or molds. They include angiosperms *(Zea mays, Pachysandra procumbens*), a protozoan ( *Sterkiella histriomuscorum*), and an “uncultured bacterium.” Comment please.

**Author response: The MG-RAST database contains multiple taxa. The hits to non-fungal species contained multiple mismatches and were deemed spurious.  We filtered the data to remove all non-fungal hits and regenerated the figures, reporting all fungal species detected with 10 reads or more.  This resulted in a higher number of species reported for some samples and fewer for others.  Figure 2 was revised and the figure legend expanded.**

5. This study revealed a surprisingly depauperate *Cannabis* foliar microbiome, compared to a recent study of *Genlisea*species, using similar methods, that identified 92 genera of organisms (Cao *et al.* 2015). See Delmotte *et al.* (2009) for rich microbiomes in other plant species. Gzebenyuk (1984) isolated 79 species of fungi from hemp stems in Russia. Comment please.

      Where are the bacteria? Much of the concern over microbiology and food safety focuses on human enteric pathogens ( *e.g., Escherichia coli, Salmonella* spp.) and opportunistic bacteria ( *e.g., Pseudomonas aeruginosa, Berkholderia cepacia*). Previous culture-based studies have isolated these organisms from *Cannabis* ( *e.g.,* Taylor *et al.* 1982, Ungerleider *et al. *1982). The only bacterium you identify (in Figure 2, erroneously identified as a yeast or mold) is *Pantoea agglomerans, *formerly known as *Enterobacter agglomerans,* a gram-negative bacterium and an opportunistic human pathogen.

**Author response: This study focuses only on the fungal microbiome.  The studies cited are looking at field-grown samples. We revised the MG-RAST figures to include all fungal species detected down to a level of 10 reads.  This reveals a significantly larger number of species in two samples. Other species display only a handful of species. Medicinal Cannabis is often grown indoors in artificial media. The first paragraph of the discussion section was expanded and split into two paragraphs as follows:**

**"**This study demonstrates detection of numerous fungal species by molecular screening of ITS2 in several dispensary-derived *Cannabis* samples. These included the toxigenic *Penicillium *species: *P. paxilli, P. citrinum*, *P. commune, P. chrysogenum, P. corylophilum, Aspergillus* species: *A. terreus, A. niger, A. flavus*, *A. versicolor *and *Eurotium repens.* In addition, a pathogenic species *Cryptococcus liquefaciens* was detected. The fungal microbiomes of the different samples differed significantly in the number and diversity of species present.  Two samples contained a large diversity of species, similar to previous studies that used field-grown samples and culture-based outgrowth methods ^2, 3, 11^. Other samples contained only a few species at significant levels. This is perhaps not surprising given the prevalence of indoor culture methods using artificial growth media for medicinal *Cannabis*. However, we do not have any knowledge of the specific growth conditions that were used for the samples analyzed.

Three different culture-based assays failed to detect all of the positive samples and one, BioLumix ^TM^, detected only one out of 7 positive samples. A review of the literature suggests that *Penicillium* microbes can be cultured on CYA media, but some may require colder temperatures (21-24C) and 7 day growth times ^40^ . Of the *Penicillium,* only *P. citrinum* has been previously reported to culture with 3M Petri-Film ^41^ . It is possible the different water activity of the culture assay compared to the natural flower environment is contributing to the false negative test results."

Minor critiques:

1.     The detection and confirmation of *Penicillium citrinum* and *Penicillium paxilli* deserves mention in the abstract of the paper! An exhaustive review of the *Cannabis* literature (McPartland *et al.* 2000) found no references to these organisms.

Here we describe the first next generation sequencing survey of the fungal communities found in dispensary based Cannabis flowers by ITS2 sequencing, and demonstrate the sensitive detection of several toxigenic *Penicillium* and *Aspergillus* species, including *P. citrinum* and *P. paxilli*, that were not detected by one or more culture-based methods currently in use for safety testing.

2.     Species names should never be capitalized. For example, *P. Citrinum* should read *P. citrinum*.  

**fixed**

3.     The full name should be spelled out the first time it appears in the Methods section, *Penicillium citrinum*.

**fixed**

4.     The Methods section should explain that the *PaxP* gene comes from *Penicillium paxilli.* The Methods section should identify NEP as New England BioLabs.

**fixed**

5.     Recommend some future directions: a comparison of indoor crops and outdoor crops (outdoor crops may show a seasonal community succession), and survey the microbiome of police seizures from well-provenanced foreign sources, such as Mexico.

**Added to conclusion paragraph.**
